# Brain-lung crosstalk: how should we manage the breathing brain?

**DOI:** 10.1186/s12890-023-02484-7

**Published:** 2023-05-23

**Authors:** Sarah Wahlster, James A. Town, Denise Battaglini, Chiara Robba

**Affiliations:** 1 Department of Neurology, University of Washington, Seattle, WA USA; 2 Department of Neurological Surgery, University of Washington, Seattle, WA USA; 3 Department of Anesthesiology and Pain Medicine, University of Washington, Seattle, WA USA; 4 Division of Pulmonary, Critical Care and Sleep Medicine, Department of Medicine, University of Washington, Seattle, USA; 5 IRCCS Ospedale Policlinico San Martino, Genova, Italy; 6 Dipartimento di Scienze Chirurgiche e Diagnostiche Integrate, Università degli Studi di Genova, Genova, Italy

## Abstract

Recent studies have drawn increasing attention to brain-lung crosstalk in critically ill patients. However, further research is needed to investigate the pathophysiological interactions between the brain and lungs, establish neuroprotective ventilatory strategies for brain-injured patients, provide guidance on potentially conflicting treatment priorities in patients with concomitant brain and lung injury, and enhance prognostic models to inform extubation and tracheostomy decisions. To bring together such research, BMC Pulmonary Medicine welcomes submissions to its new Collection on ‘Brain-lung crosstalk’.

## Background

Patients with acute brain injury (ABI) constitute up to 25% of patients requiring mechanical ventilation (MV) [1]. However, there is scarce evidence to guide ventilatory targets in this population. Appropriate management of MV in ABI should account for some unique, clinically relevant considerations: Cerebral and pulmonary pathophysiology are intricately intertwined via complex, often bi-directional pathways, which are not yet fully understood. Different arterial blood gas targets may be required in select patients to minimize secondary ischemic brain injury, optimize intracranial pressure (ICP), or enhance cerebral perfusion. Lung protective ventilatory strategies established in the general critical care population may conflict with therapeutic strategies aimed to protect the brain. Furthermore, there are substantial uncertainties around optimal ventilator liberation strategies, predicting extubation success and the ideal timing and need for tracheotomy in brain-injured patients. All together, these challenges result in marked practice variations worldwide. Therefore, more research is required to establish optimal standards of care in this population.

Experimental research and clinical studies have established complex, often bi-directional, pathways between the brain and lungs. ABI has been shown to precipitate lung injury and modulate pulmonary physiology via several mechanisms, including elevated ICP, systemic inflammatory response, hormonal dysregulation, catecholamine surges and dysregulated central breathing control [2]. Conversely, arterial blood gas derangements and systemic inflammation can precipitate secondary brain injury. Long-standing cognitive deficits and mood disorders are frequently encountered after acute respiratory distress syndrome (ARDS), even in the absence of known previous ABI. In critically ill patients, common complications such as hypotension, shock, sedation, polypharmacy, fever, and delirium may also contribute to these interactions. Further establishing pathophysiology and key mechanisms generating this crosstalk may help identify preventive measures and therapeutic targets.

A recent European Society of Intensive Care Medicine (ESICM) consensus statement acknowledges uncertainties and paucity of evidence regarding ventilator targets and parameters for patients with ABI [3]. The optimal arterial partial pressure of oxygen (P_a_O_2_) and partial pressure of carbon dioxide (P_a_CO_2_) ranges remains to be established in this population. While both hypoxemia and hyperoxemia are detrimental, the exact margins of the u-shaped curve and individualized P_a_O_2_ thresholds are yet to be determined. The value of brain tissue oxygen (PbtO_2_) guided management is currently being investigated: A phase II randomized trial found that a protocolized approach based on ICP and P_b_tO_2_ resulted in less duration of brain hypoxia and a trend towards lower mortality among patients with severe traumatic brain injury, [4] and further studies are underway. P_a_CO_2_ is thought to be a critical parameter in ABI as a key mediator of cerebral blood flow: Hypercarbia may contribute to cerebral vasodilation and elevated ICP, and hypocarbia may cause cerebral vasoconstriction and ischemic brain injury. The previously established practice of therapeutic hyperventilation has become increasingly controversial due to concerns about decreased cerebral perfusion and rebound intracranial hypertension. Meanwhile, the potential benefits of targeted therapeutic P_a_CO_2_ ranges in specific ABI subpopulations are being studied.

Recent studies have assessed the utilization of various ventilator parameters and associations with outcomes in ABI. Specifically, higher tidal volumes, driving pressure, respiratory rate, and mechanical power have been associated with worse outcomes [5, 6]. Results of the VENTIBRAIN study, the largest observational prospective multicenter trial to date examining MV practices in ABI, are forthcoming [7]. Many of these studies demonstrate substantial practice variations, highlighting a need for more research to guide the standardization of practices. While these investigations indicate a growing interest and progress in this area, they are largely limited due to their retrospective or observational design. Randomized studies are needed to assess the causality of these findings, and future research to compare thresholds for ventilatory settings between ABI and other critical care populations is warranted.

With limited data to guide ventilator management in ABI, clinicians might find themselves conflicted when managing patients with concomitant brain and lung injury. ARDS is common in critically ill patients with ABI and is associated with poor outcomes. However, landmark ARDS studies have excluded patients with neurological disease, specifically those with elevated ICP. A main concern in patients with ABI and ARDS is the risk of ICP elevations due to lung protective ventilation, higher levels of positive end-expiratory pressure (PEEP), or prone positioning (PP). While utilization of these strategies may be harmful in specific ABI subpopulations, these concerns may also prompt clinicians to withhold well-established therapies with proven benefits. A recent study demonstrated increased utilization of lung protective ventilation from 2004 to 2016 in ABI, [8] however, only 53% of clinicians in an ESICM survey reported using 4–6 ml/kg/PBW for patients with ABI and P_a_O_2_/F_i_O_2_ ratio < 150 [9]. A recent, randomized trial of 30 patients showed no substantial effect of lung protective ventilation on ICP or cerebral autoregulation in most patients, although 22% required interruptions of the protocol for sustained ICP elevations [10]. Studies investigating the effect of PEEP on ICP show mixed results, with some suggesting that ICP and cerebral perfusion pressure (CPP) may be driven by PEEP-dependent decreases in cardiac output and mean arterial pressure, or that PEEP-mediated ICP elevations are mostly observed in patients with poor respiratory compliance. Studies assessing ICP and CPP changes with PP are limited by small size, heterogenous populations, short proning duration, and lack of long-term neurological outcomes. Overall, most studies did demonstrate substantial ICP elevations and decrease in CPP with PP, but also showed significant improvement of P_a_O_2_ and P_b_tO_2_ [11]. Future research is needed to establish long-term effects as well as identify which ABI subpopulations may be at higher risk for clinically relevant ICP crises. The role of invasive and non-invasive neuromonitoring in identifying individualized targets and guiding ICP treatments also warrants further investigation in this population.

Last, there is a need for more prognostic clarity to inform extubation and tracheostomy decisions. Patients with ABI commonly require MV due to decreased level of consciousness and impaired airway protective reflexes, and our ability to predict extubation outcomes remains poor in this population. A growing body of research has identified factors associated with extubation success in ABI. The recently published prospective ENIO trial, which included 1512 patients from 73 centers across 18 countries, showed a 19% extubation failure rate within 5 days and substantial practice variations between countries [12]. An extubation success prediction model including 20 factors was developed based on this dataset’s accuracy, but the limited accuracy of this score heralds that the task of correctly predicting extubation success remains a major challenge. There is similar uncertainty about the indication and ideal timing of tracheostomy placement. While the data regarding tracheostomy timing in ABI have shown conflicting results overall, a recent large meta-analysis [13] and a multicenter, randomized controlled trial (SETPOINT-2) [14] in mixed stroke populations found no benefits of early tracheostomy within five days, and 22% of patients randomized to the late tracheostomy group were able to successfully wean from MV and did not require tracheostomy placement. In addition to our limited ability to anticipate extubation success, substantial uncertainties about long-term neurological prognosis often complicate the decision to pursue tracheostomy as a life-sustaining treatment. More research is needed to better guide the goals of care conversations and end-of-life decisions with our patients’ families.

In light of these ongoing questions, BMC Pulmonary Medicine has launched a Collection to bring together research on “Brain-lung crosstalk” to highlight new findings regarding the pathophysiological interactions between the brain and lungs, inform MV strategies in ABI, provide guidance on navigating brain-lung conflict, and enhance prognostic models to inform extubation and tracheostomy decisions. To help further our understanding of cross-talk between the brain and lungs, we aim to promote innovative research from diverse backgrounds, including basic, translational, and clinical science.

## Data Availability

Not applicable.

## Abbreviations

ABI: acute brain injury
ARDS: acute respiratory distress syndrome
ENIO: Extubation Strategies in Neuro-Intensive Care Unit Patients and Associations with Outcome
ESICM: European Society of Intensive Care Medicine
CPP: cerebral perfusion pressure
ICP: intracranial pressure
LPV: lung protective ventilation
MV: mechanical ventilation
PEEP: positive end-expiratory pressure
SETPOINT: Stroke-related Early Tracheostomy versus Prolonged Orotracheal Intubation in Neurocritical Care Trial
